# History of a Case of Autumnal Fever; Accompanied by the Vomiting of a Fluid of a Deep Blue Color

**Published:** 1841-05

**Authors:** George K. Holloway

**Affiliations:** Panola, Mississippi


### Art III.—*History of a Case of Autumnal Fever; accompanied by the vomiting of a fluid of a deep blue color.* By George K. Holloway, M. D., of Panola, Mississippi.

On the 19th of September, 1840, J. W. Mitchell, aged 22 years, of a bilious temperament and sedentary habits, began to complain of lassitude, and pain in his head and back, with confined bowels. These symptoms increased through the night, and on the 20th he was much worse than the day before. The following night was one of great suffering and restlessness; the pain having extended from the spine into his limbs and epigastrium, the latter of which, moreover, was the seat of oppression and fullness. The next morning, 21st., his visage was haggard, and, spontaneous vomiting coming on, he threw up a quantity of *thick, deep blue fluid.* The lady of the house at which the patient lodged, being struck with its appearance, washed a rag upon which he had vomited, in clear water, and found that it imparted an indigo color to the fluid. The master of the house now administered a dose of colomel which opened his bowels, but did not mitigate his symptoms. Indeed, they increased in violence, and a sense of choaking with frequent sighs came on, the material vomited up, still exhibiting the same color. On the 22d his condition was still worse, as he had intense thirst and great restlessness, and more frequent blue vomitings. Another and larger dose of calomel was now administered, which produced copious, thin, bilious stools, with considerable discharges of flatus, but the succeeding night was one of great sleeplessness and great anxiety.

In the forenoon of the next day, 23d, we saw him for the first time. He was in a state of the utmost alarm; his visage almost Hippocratic; eyes red and wild; forehead, neck and breast, cool and wet with sweat; tongue tremulous, pointed towards the tip, dry and red on its edges, its papillæ of a darker hue and raised, and it was with difficulty that he could project it over his teeth; the mucous lining of his mouth and fauces were as red as in scarlatina; his pulse was 120 or more in a minute, but full, soft and compressible; his restlessness was extreme; the pain in his loins intense; the discharge of urine small and with difficulty; the discharges from the bowels watery, bilious and frequent; the epigastric distress intense; and the vomiting of a deep blue matter constant. This fluid stained a linen rag as blue as if it had been dipped into a saturated solution of Indigo. Its quantity was very great, and the stomach so excited, that the slightest mechanical irritation of the fauces would bring it up. Not having before met with such a case, we supposed the patient might have taken something which had colored the contents of his stomach; but on careful inquiry found that he had not.

The case appeared hopeless, but we felt it a duty to prescribe, and directed as follows: A blister from the ensiform cartilage to the symphisis pubis, and another on each ankle, with one of the following powders every four hours—

℞. Submur. Hydrarg. grs. xv.
Pulv. Ipecac et Opii aa grs. viij.

Mix and divide into four powders.

To the first of these powders we added a quarter of a grain of the sulphate of morphine. The powders did not increase his nausea, which, at no time, was proportionate to the vomiting; and they moderated the purging. Towards day of the twenty-fourth, he had a tolerably consistent alvine evacuation, which, however, displayed a bluish tint, and was extremely fœtid. We now omitted the ipecac and opium, and gave him three grains of calomel with a fourth of a grain of the sulphate of morphine every four hours. During the day he had two consistent alvine discharges.

On the 25th we found that he had passed the night without any further alvine evacuation, but the vomiting had continued. His pulse was less frequent, and rather fuller. Same medicines continued.

26th. Had one evacuation last night, after which he was inclined to sleep. This morning he seemed to us better; but complained of some abdominal fulness. We prescribed five grains of calomel, and half a grain of morphine, every six hours. In the course of the day, he had one full and consistent stool, of a bluish green colôr. In the evening we left him on the use of this compound, with orders that he should take a teaspoon full of denarcotised laudanum, if excessive and watery purging should come on.

27th. On arriving we learned that in the early part of the night he had a most profuse evacuation, followed by great relief, and another also large, and likewise productive of relief. Immediately after the last, his attendants unfortunately gave him the laudanum. He had no more, and did not sleep. Our arrival was at 10 o'clock, when we found him in all respects much worse. A hiccup had supervened, with subsultus tendinum and tinnitus aurium; the surface of his body was covered with a cold sweat; his respiration was hurried and laborious, with constant sighing, and ceaseless jactitation; he vomited much, but the fluid now thrown up was of a bistre brown instead of a blue color; his pulse had also suffered a signal change, being very small and frequent, and his alarm and terror were of the most remarkable kind. In this extremity we made extensive application of blisters; gave him twelve grains of calomel every three hours, resorted to injections and suppositories to bring on immediate alvine evacuation, and administered stimulants and tonics; but the last increased the irritability of his stomach and had to be omitted. About 8 P. M. he had a copious, tolerably thick, but fœtid alvine discharge, which was followed by great relief from most of his symptoms, and a slight strangury came on.

Some of his friends now expressed to him the opinion that his disease would terminate fatally, whereupon his terror, which had much abated, was reproduced, and all his bodily symptoms became worse. His pulse sunk, as did the heat of his extremities; his face assumed a cadaverous aspect; the vomiting of the bistre-brown fluid, as thick as the white of egg, returned, and dissolution seemed to be at hand.

A consulting physician was now sent for, but did not arrive till the morning of the 28th. Before sunrise of this morning, our patient threw up about two quarts of fluid of the same color as that discharged the day before, and he continued to gulp up the same kind of fluid till within fifteen minutes of his dissolution, which, however, soon occurred. A *post mortem* examination was not made.

We regard this as a case of malignant Congestive Fever, the striking peculiarity of which was the blue and brown discharge from the stomach.

For our views on Congestive Fever, we beg leave to refer to the *South. Med. and Surgical Journal*, for the month of May or June, 1839, published at Augusta, Georgia. Those views, however, were formed from observations made in the arid climate of that state, and are not in all respects applicable to the fever as it occurs in the more humid atmosphere of the state of Mississippi. Here the symptoms are more insidious and equivocal than in Georgia. Often to superficial observation, nothing more than a simple intermittent of the tertian or quotidian type, or a mild remittent, seems to be indicated; but a close observer will detect its malignant character, in the early cold and clammy sweats of the upper part of the body; the tremulous motion of the tongue; its conical point; the difficulty of projecting it; the dry and rough, or smooth and slimy surface which it presents, and the fiery redness of its edges—appearances which do not occur in simple intermittents and remittents, except under excessive purging. Great flatulence is also present in the malignant cases, and the intermission is, in general, imperfect.

The only modification of the treatment we had pursued in Georgia, which we have found necessary in Mississippi, is the administration of larger doses of cathartic medicines in the beginning, as more is required to purge the patient; but afterwards we have found smaller portions, than are required in Georgia, sufficient to keep up the purging; which is more likely to degenerate into a watery diarrhœa in the former than in the latter region. In both states, our reliance is on calomel, ipecac, and opium, or the sulphate of morphine. Saline and drastic purgatives are injurious, and the lancet generally inadmissible.

*October*, 1840.

